# Case report: Resection of a massive primary sacrococcygeal mature teratoma in an adult using 3-dimensional reconstruction and mixed reality technology

**DOI:** 10.3389/fsurg.2022.948388

**Published:** 2022-09-22

**Authors:** He Zhang, Lu Ji, Jinxin Liu, Shizhe Li, Ting Chen, Jiatong Li, Guanning Shang

**Affiliations:** ^1^ Department of Bone and Soft Tissue Oncology, Department of Surgery, Shengjing Hospital, China Medical University, ShenYang, China; ^2^ Department of Gynecology and Obstetrics, Shengjing Hospital, China Medical University, ShenYang, China

**Keywords:** mixed reality, pseudoarthrosis, teratoma, sacrococcygeal, three-dimensional reconstruction (3D reconstruction), case report

## Abstract

**Introduction:**

Teratomas are rare neoplasms that arise from pluripotent germ cells. Sacrococcygeal teratomas are often diagnosed in infants but are rare in adults; a mature teratoma can contain hair, teeth, bony tissue, and other mature tissue types. Herein, we report for the first time a patient with a teratoma containing intact bones that formed a pseudoarthrosis.

**Case report:**

A 49-year-old woman was admitted to hospital after a massive life-long sciatic tumor had begun to grow larger over the past year. A 16 cm × 25 cm solid mass with a clear boundary was palpable in the sacrococcygeal region. Radiography, computed tomography, and magnetic resonance imaging indicated a sacrococcygeal teratoma, although blood alpha-fetoprotein levels were normal. The teratoma was completely excised using 3-dimensional reconstruction mixed reality (MR) technology with no notable complications. Postoperative pathological examination of the excised lesion confirmed a mature teratoma. Interestingly, two intact irregular bones that formed a pseudoarthrosis were isolated; one was 11 cm and the other 6 cm. The patient is currently healthy and has experienced no recurrences.

**Conclusion:**

Sacrococcygeal teratomas are rare, especially in adults, and often comprised lots of components, such as fat, bony tissue. However, it's first reported that formation of pseudoarthrosis in this case so far. It is difficult for surgeons to achieve complete excision without complications owing to the complex anatomic structure of the sacrum. The 3-dimensional reconstruction and mixed reality (MR) technology based on computed tomography can provide spatial visualization, which allows surgeons to examine the teratoma at different angles preoperatively. Combining 3-dimensional reconstruction and mixed reality (MR) technology in this case facilitated complete resection and prevented recurrence.

## Introduction

Teratomas are rare congenital tumors derived from germ cells; they account for 2.5% of all germ cell tumors (1) and are commonly diagnosed in infants and children but rarely in adults (2). Outside the gonads, teratomas are most commonly diagnosed in the midline regions of the body (3). These tumors can be benign or malignant depending on their differentiation levels (4); a high differentiation level and abundant mature tissue component are often found in benign teratomas, whereas malignant tumors tend to exhibit low differentiation and immature tissues (5). Three-dimensional (3D) reconstruction and mixed reality (MR) are advanced surgical technologies that play important roles in precision medicine. The former can transform two-dimensional images into 3D models, allowing surgeons to observe the complex anatomical structures of the tissues surrounding the tumor as well as the tumor-environment relationship. The latter provides an interactive experience based on the 3D virtual image, allowing surgeons to simulate the surgery and verify the procedural plan preoperatively.

In this report, we describe a rare sacrococcygeal teratoma diagnosed in an adult that contained 2 intact bones forming a pseudoarthrosis. We also describe how 3D reconstruction and mixed reality (MR) technology were used to ensure precise surgical resection. This report was prepared according to the SCARE checklist (6).

## Case presentation

A 49-year-old woman presented at Shengjing Hospital (affiliated with China Medical University) after noticing that a massive sacrococcygeal tumor she had since birth had been growing over the past year. One month before her visit, the patient felt pain in her hip owing to a rapidly enlarging mass. The patient didn't receive any medical intervention and the symptoms were not released over past year. The patient was previously healthy with no history of chronic cardiovascular diseases, infectious diseases, allergy, or surgery. She did not smoke or consume alcohol, and had no familial diseases.

**Figure 1:**
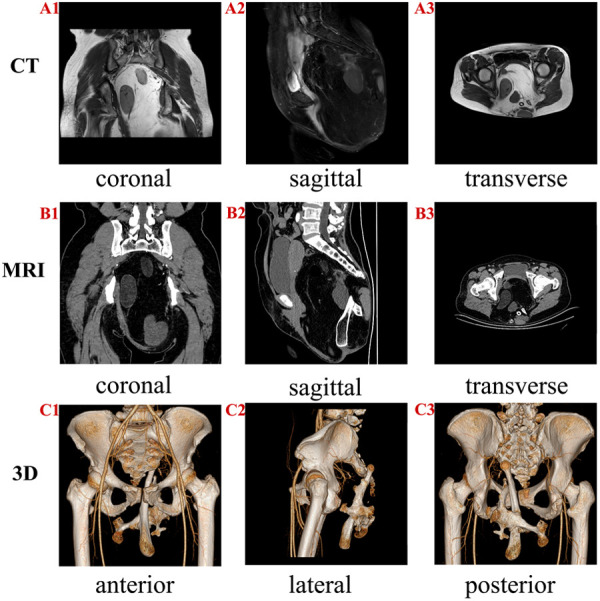
Magnetic resonance imaging (MRI) and 3D computed tomography (CT) of the patient. (**A1–A3**) CT showing different views of the teratoma. A huge space-occupying lesion exhibiting different densities can be observed in the pelvic cavity. A clear boundary is observed between the teratoma and pelvis. (**B**1**–B**3) MRI showing various views of the teratoma. The massive sacrococcygeal multiloculated lesion comprises a large amount of fat, sebaceous material, and long bone. (**C**1**–C**3) Various 3D views of the bone reconstruction based on CT; the 3D reconstruction allowed the visualization of the spatial positions of the bones within the teratoma as well as the lesion's relationship with the pelvis.

Physical examination revealed a palpable 16 cm long × 25 cm wide solid mass in the sacrococcygeal region, with a clear boundary and good mobility. There was an obvious osteal protuberance in the center of the mass that had poor mobility. Preoperative laboratory workups including complete blood count, urinalysis, liver function test, and renal panel were within normal range. Pelvic radiography revealed a long rod-like bone in the pelvic cavity, while pelvic enhanced computed tomography (CT) revealed a massive space-occupying lesion in the pelvic-sacrococcygeal region that was 14.5 cm × 10.5 cm × 21.5 cm. Pelvic enhanced magnetic resonance imaging revealed a massive sacrococcygeal multiloculated teratoma comprised mainly of a fatty, sebaceous material as well as a long bone lacking any obvious abnormalities on enhanced scanning (Figure 1).

**Figure 2:**
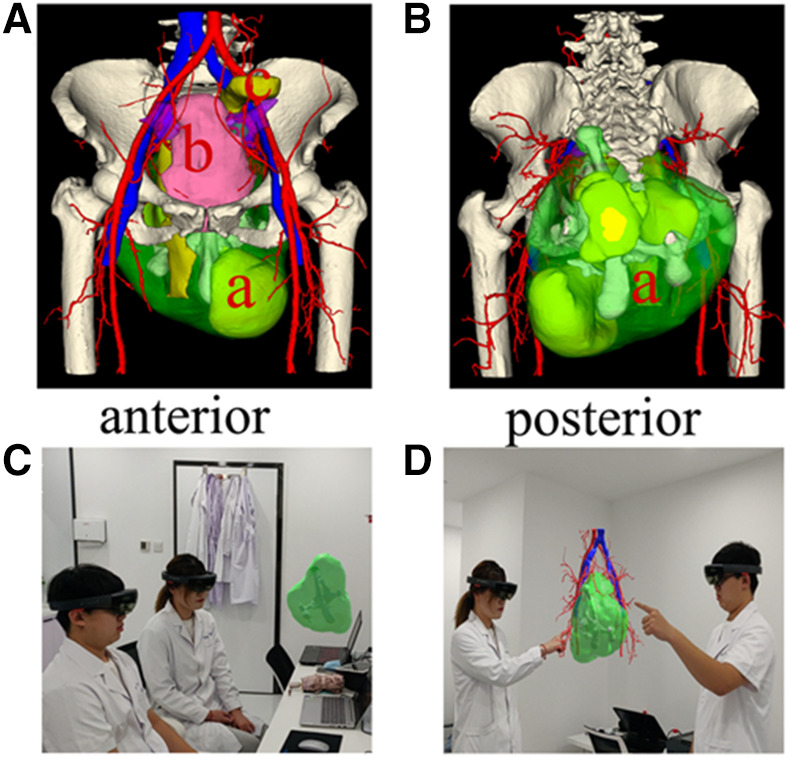
The application of 3D reconstruction and mixed reality (MR) technology in our patient. (**A,B**) The 3D reconstruction images accurately displaying the relationship between the teratoma and surrounding tissues through various angles. (a) teratoma; (b) bladder; (c) rectum. (**C,D**) mixed reality (MR) technology showing the spatial location of the teratoma and its orientation with respect to the pelvic cavity. This technology was used to devise a surgical strategy and simulate the surgical process.

A multidisciplinary consultation was arranged to decide on the optimal therapeutic approach based on 3D reconstruction and mixed reality (MR) technology, which were used to ensure precise surgical resection. The 3D reconstruction was performed using the “visual volume” software (Shenyang, China) with CT-derived data. The 3D model was then uploaded into the mixed reality (MR) image system, whereupon the surgeon could visualize the 3D model through the mixed reality (MR) helmet (Microsoft Corp, Redmond, WA, United States) (Figure 2). This enabled to discern the relationship between the teratoma and surrounding tissues (such as vessels and nerves) directly. Furthermore, the mixed reality (MR) visual software allowed the operator to move, rotate, hide, and delete elements of the 3D model, thereby allowing him to simulate the surgery and eliminate potential errors. The multidisciplinary team had concluded that the lesion was likely a sacrococcygeal teratoma, and that its final diagnosis ought to be verified *via* postoperative pathological analysis.

The surgery was performed on March 26, 2021. After administering general anesthesia and disinfecting the area, a 40 cm arced incision was created in the sacrococcygeal region from the posterior superior iliac spine to the contralateral posterior superior iliac spine along with the lower boundary of the tumor (i.e., resembling a smiley face). An intact encapsulated mass was found under the deep fascia; its upper boundary reached the sacral 5 vertebra, its lower boundary reached the anococcygeal ligament, and its bilateral boundaries were adjacent to the gluteus and surrounding soft tissue. The tumor protruded into the pelvic cavity and compressed the ampulla recti. Given that it had an intact capsule, the tumor did not invade the surrounding organs and we decided to remove the teratoma integrally to decrease the recurrence rate. The teratoma was rigid and lacked elasticity because of the two bones inside, which may break the capsule leading to incomplete resection if the surgical area was small. Therefore, we removed the coccyx to expose an enough space for surgical operation. Next, we separated the tumor from the pelvic cavity with the guidance of 3D reconstruction and mixed reality (MR) technology. Especially, when separating the deep bottom of the teratoma, meticulous operation was necessary to avoid bladder and rectum injury. Finally, we reconstructed the sacral structure to help recover sciatic function by anchoring a mesh through the suture to the surrounding tissue in order to provide mechanical support. Muscle reconstruction was achieved by suturing the anococcygeal ligament to the coccygeal stump (Supplementary Figures). Surgery was completed with no complications.

**Figure 3:**
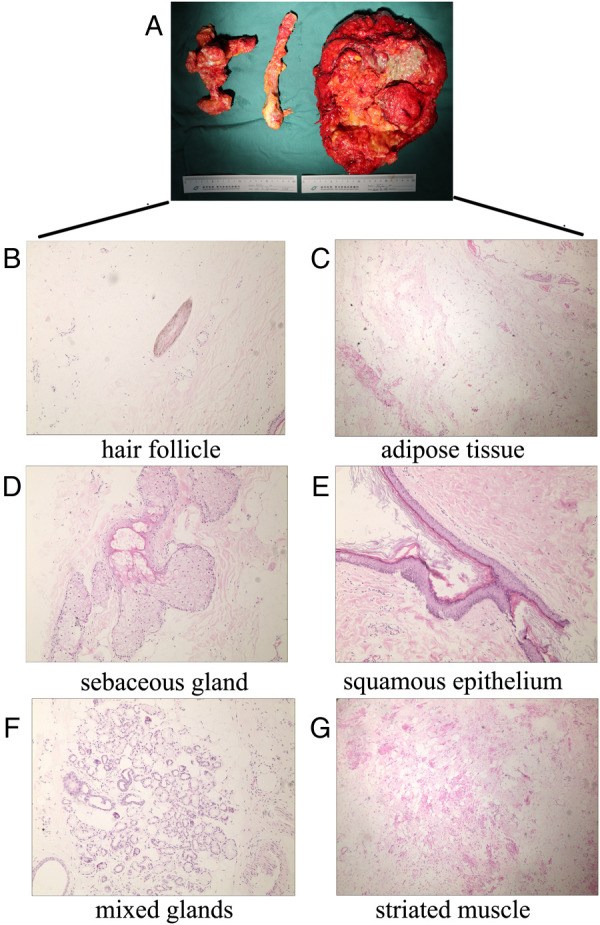
The gross appearance and pathological examination of the teratoma. (**A**) The teratoma measured 24 × 23 × 15 cm and weighed 5 kg. Two intact bones were isolated from the mass: one was 11 cm long with a diameter of 3 cm, and the other was irregular (6 × 6 × 5 cm). The two bones formed a pseudoarthrosis. (**B**–**G**) Pathological slides stained with hematoxylin and eosin: **(B**) hair follicle, (**C**) adipose tissue, (**D**) sebaceous gland, (**E**) squamous epithelium, (**F**) mixed glands, and (**G**) striated muscle.

The excised specimen was transferred to the Pathology Department for cytological diagnosis. Macroscopically, it was 24 cm × 23 cm × 15 cm, and weighed 5 kg. The surface of the mass was capsulated an*d* bosselated. The cut surface exhibited multiple cysts that contained yellow fat, gray viscous sebaceous matter, hair, muscle, and bone tissue. Of particular interest, two intact irregular bones that formed a pseudoarthrosis were isolated from the mass; one was 11 cm long and the other 6 cm (Figure 3A). Microscopically, fibrous squamous epithelium with cutaneous appendages, hair follicles, sebaceous glands, bony trabeculae, and fat tissue was observed in the section (Figures 3B–G). There were no immature neural or embryonic tissues (which are markers of malignant transformation) in the section. The final diagnosis was a mature sacrococcygeal teratoma.

**Figure 4:**
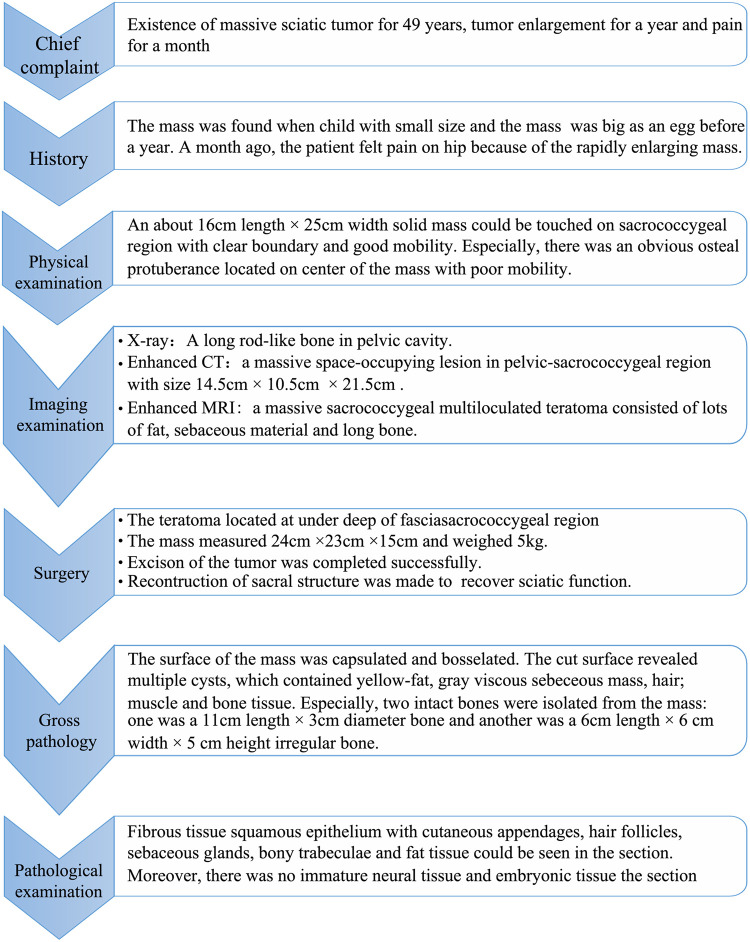
The therapeutic course of the patient. The current case report is presented based on this sequence of events.

The postoperative routine blood tests were drawn every 3 days. The labs showed a slight elevation of white blood cell count, C-reactive protein level, and erythrocyte sedimentation rate, which were appropriate for a normal physiological response postoperatively. She was discharged on postoperative day 7 with no complications. Her treatment course was summarized in Figure 4.

## Follow-up and outcomes

The patient was followed up in our clinic every 6 months after discharge to monitor for local recurrence. Physical exams, AFP, ultrasound, and pelvic MRI were performed during each clinic visit. At 1-year follow-up, her AFP remained normal, and surveillance imaging (ultrasound and MRI) didn't show any signs of recurrence. In the perspective of the patient, the symptoms including pain, disability of defecation and urination was released entirely. The life quality of the patient was significantly improved. Meanwhile, the patient satisfied with the successful surgery and uneventful recovery. She held the view that she was in good condition and enjoyed a happy life like normal person.

## Discussion

Teratoma is a rare type of neoplasm that originates from primordial germ cells; its site of occurrence varies with age (7). In infants, teratomas are often discovered in the sacrococcygeal region, whereas adolescents and adults tend to have such tumors diagnosed more commonly in the gonads (5). These tumors can be divided into two types, mature and immature. Mature teratomas are often detected in adults and comprise well-differentiated components, although they tend to undergo malignant transformation (5); meanwhile, immature teratomas are more frequently found in infants and children, and exhibit partial differentiation (8). Sacrococcygeal teratomas more commonly occur in infants than in adults (9, 10). Hence, our patient's teratoma is rare not only because of its discovery in the sacrococcygeal region but also because it was diagnosed in an adult 49-year-old woman.

Sacrococcygeal teratomas can be classified into four types according to their depth of infiltration; these range from type I tumors that are located outside the sacrococcygeal region to type IV lesions that exist exclusively in the presacral region and pelvic cavity (11). Our patient's teratoma was categorized as type III, which typically entails a complicated surgery.

Surgery is the optimal treatment for sacrococcygeal teratoma (12), the planning of which requires taking two important aspects into account. One is achieving complete resection to avoid recurrence, and the other is to prevent complications as much as possible. A study showed almost 11 percent of patients recurred in 3 years postoperatively due to incomplete resection (13). Traditionally, more complete resections are associated with greater rates of surgical complications including urinary tract and bowel dysfunction, hemorrhage, and nerve injury (14); therefore, attaining the optimal balance between maximum therapeutic effect and minimal surgical complications requires surgeons to devise a definitive surgical strategy *via* thorough analysis of the imaging data. In our patient, 3D reconstruction and mixed reality (MR) technology were used to strategize for and navigate the procedure (15). With 3D reconstruction, the spatial structure and relationship between the teratoma and surrounding tissues could be displayed (16). Mixed reality (MR) technology was used to transform the 3D model into a spatial virtual image; its greatest advantage was its interactive function, given that we were able to discern the relevant anatomic structures better. In fact, we were able to simulate the surgical procedure to ensure its effectiveness *via* this interactive system. Furthermore, the two bones formed a pseudoarthrosis that meant a rigid structure within the teratoma. The rigid structure may be an obstacle to surgery, and we should ensure enough surgical incision to remove the teratoma from pelvic cavity. 3D reconstruction and mixed reality (MR) technology allowed to predict related intra-operative challenges, assisted the surgeons in devising a meticulous surgical plan to prevent unnecessary damage, and ensured complete tumor removal. Moreover, visualization aided us in explaining the planned procedure to the patient, who was thus able to understand both the surgical process and associated risks (17). We successfully removed our patient's teratoma and coccyx without damaging the gluteal artery, sciatic nerve, or rectal ampulla owing to our use of 3D reconstruction and mixed reality (MR) technology.

However, there are some limitations in terms of using 3D reconstruction and mixed reality (MR) technology in clinical practice. The spatial anatomic structure that mixed reality (MR) provides is based only on CT-derived values; in actuality, the anatomic structure may be more complicated in a narrow surgical view, and may be affected by hemorrhage and other factors (18). Mixed reality (MR) technology is also limited by the resolution of the CT; some small tissues may not be detected, which in turn may lead to hemorrhage, organ injury, nerve injury and other surgical complications.

Postoperative monitoring is also necessary (19), as the possibilities of recurrence and malignant transformation exist. The probability of malignant transformation reportedly increases with age (20); as such, regular CT and physical examinations should be performed to detect any progression in residual teratoma tissue over the course of the patient's life (20). To that end, alpha-fetoprotein is a sensitive serum marker that can be used to screen for teratoma recurrence, as well as the severity thereof (21).

## Conclusion

Sacrococcygeal teratomas rarely occur in adult women. Interestingly, formation of pseudoarthrosis in the teratoma is first reported in this case. Given the risks of surgical complications and incomplete excision with these tumor types, 3D reconstruction and mixed reality (MR) technology are advanced auxiliary tools that surgeons can use to achieve satisfactory outcomes.

## Data Availability

The original contributions presented in the study are included in the article/**Supplementary Material**, further inquiries can be directed to the corresponding author/s.
