# Supplementary figures and images for: Case report: Resection of a massive primary sacrococcygeal mature teratoma in an adult using 3-dimensional reconstruction and mixed reality technology
Source: Front Surg. 2022 Sep 22;9:948388. doi: 10.3389/fsurg.2022.948388 (PMC9632992; doi:10.3389/fsurg.2022.948388)

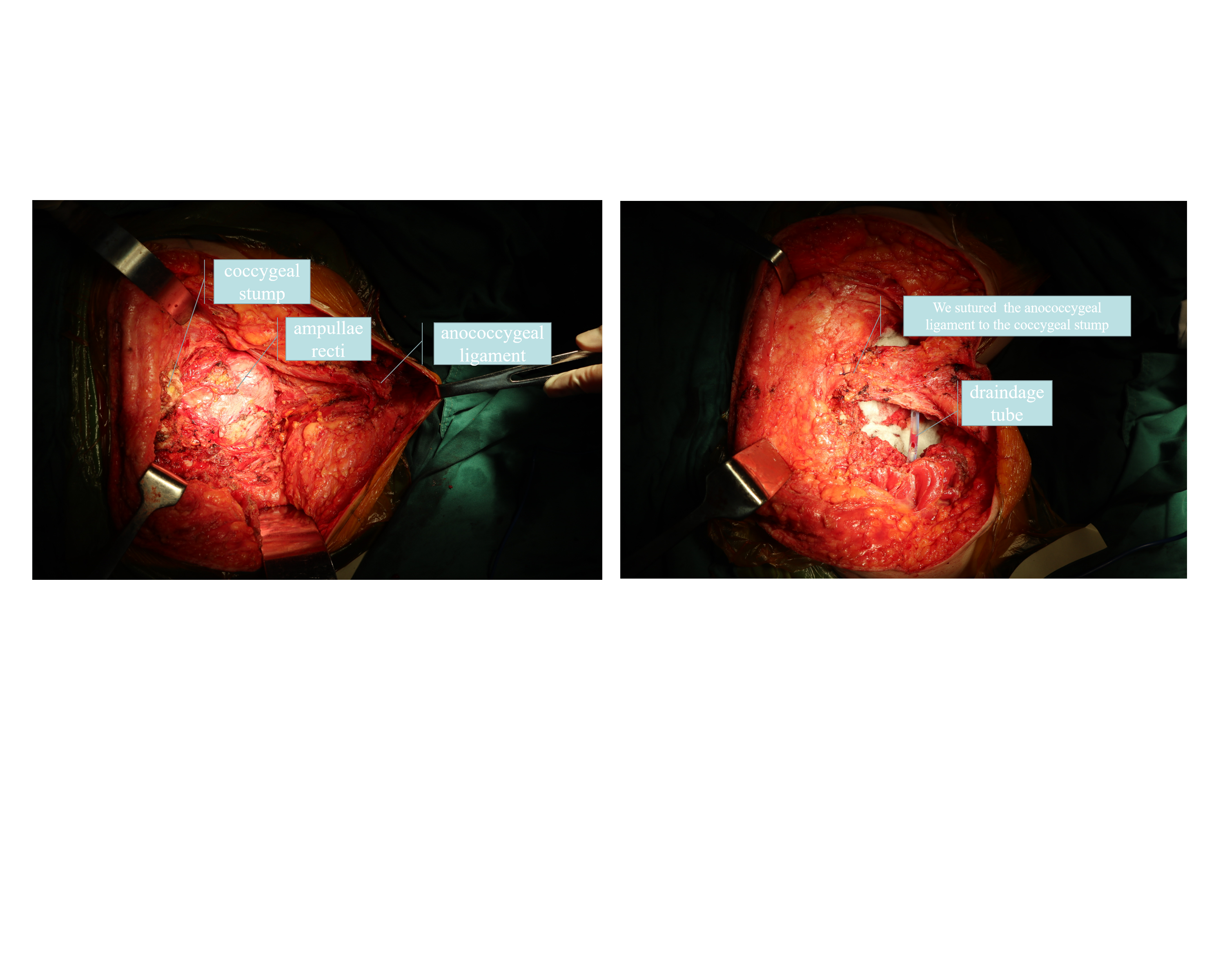

Supplement: Supplementary file 1 [file Image1.tif]
